# Zinc Oxide Nanoparticles and Their Application in Adsorption of Toxic Dye from Aqueous Solution

**DOI:** 10.3390/polym14153086

**Published:** 2022-07-29

**Authors:** Wafa Shamsan Al-Arjan

**Affiliations:** Department of Chemistry, College of Science, King Faisal University, P.O. Box 400, Al-Ahsa 31982, Saudi Arabia; walarjan@kfu.edu.sa

**Keywords:** Ismate violet 2R dye, IV2R, ZnO-NP, BET, kinetics, isotherm modelling, thermodynamic, adsorption

## Abstract

Dye waste is one of the most serious types of pollution in natural water bodies, since its presence can be easily detected by the naked eye, and it is not easily biodegradable. In this study, zinc oxide nanoparticles (ZnO-NPs) were generated using a chemical reduction approach involving the zinc nitrate procedure. Fourier transform infrared (FTIR), scanning electron microscopy (SEM), Brunauer-Emmett-Teller (BET), and UV-vis techniques were used to analyse the surface of ZnO-NPs. The results indicate the creation of ZnO-NPs with a surface area of 95.83 m^2^ g^−1^ and a pore volume of 0.058 cm^3^ g^−1^, as well as an average pore size of 1.22 nm. In addition, the ZnO-NPs were used as an adsorbent for the removal of Ismate violet 2R (IV2R) dye from aqueous solutions under various conditions (dye concentration, pH, contact time, temperature, and adsorbent dosage) using a batch adsorption technique. Furthermore, FTIR and SEM examinations performed before and after the adsorption process indicated that the surface functionalisation and shape of the ZnO-NP nanocomposites changed significantly. A batch adsorption analysis was used to examine the extent to which operating parameters, the equilibrium isotherm, adsorption kinetics, and thermodynamics affected the results. The results of the batch technique revealed that the best results were obtained in the treatment with 0.04 g of ZnO-NP nanoparticles at 30 °C and pH 2 with an initial dye concentration of 10 mg L^−1^, which removed 91.5% and 65.6% of dye from synthetic and textile industry effluents, respectively. Additionally, six adsorption isotherm models were investigated by mathematical modelling and were validated for the adsorption process, and error function equations were applied to the isotherm model results in order to find the best-fit isotherm model. Likewise, the pseudo-second-order kinetic model fit well. A thermodynamic study revealed that IV2R adsorption on ZnO-NPs is a spontaneous, endothermic, and feasible sorption process. Finally, the synthesised nanocomposites prove to be excellent candidates for IV2R removal from water and real wastewater systems.

## 1. Introduction

In terms of industrial pollution, textile industries are a major source of pollution since they use a lot of water and chemicals and discharge high levels of toxic and non-biodegradable dye effluents [1,2]. Dyes are widely applied in a variety of textile-based industries due to their advantageous properties [3,4], such as bright colours, water resistance, and ease of application [5]. Several sectors, including textile dyeing (60%), paper (10%), and plastic materials (10%), utilise large amounts of synthetic dyes (10%). According to some reports, there are approximately 100,000 commercially accessible dyes with a production capacity of more than 7 × 10^5^ metric tons per year, with the textile industry using about 10,000 compounds [6]. Dye industry effluents, in particular, necessitate not only the treatment of problematic wastewater with high chemical and biological oxygen demands, suspended particles, and hazardous chemicals but also the treatment of dyes that are perceived by human eyes at very low concentrations [5]. When dyes are released into receiving water bodies, they form hazardous amines through reductive cleavage of azo linkages, which can harm essential organs such as the brain, liver, kidney, central nervous system, and reproductive system. Furthermore, synthetic dyes may have an unfavourable effect on some aquatic life’s photosynthetic activity due to the presence of aromatics, metals, chlorides, and other contaminants. Therefore, as a consequence, their removal from aquatic environments is important and the target of numerous scientific studies [7,8,9]. Chemical precipitation [10], flocculation/coagulation [11,12], membrane technology [13], oxidation technology [14], electrolytic reduction [15], ion exchange [16], and biological adsorption [17,18,19,20] are developed methods for dyes and pollutant removal from water/wastewater. Recently, the adsorption process has been one of the most commonly used procedures for dye removal due to its simplicity and high efficacy, as well as the suitability of the use of a wide range of adsorbents [21,22,23]. Moreover, various nanoparticles have been investigated for dye adsorption due to the ease of changing their surface functionality and their high surface-to-volume ratio for increased adsorption capacity and efficiency [24]. Nanosized metal oxides, such as nanosized manganese oxides, ferric oxides, aluminium oxides, magnesium oxides, and cerium oxides, are thought to be capable of adsorbing dyes from aqueous solutions [25]. Additionally, these nanoparticles have been intensively investigated as extremely efficient absorbents for the removal of heavy metal ions from water and wastewater. They have a number of advantages, including high ability, unsaturated surfaces, ease of use, quick kinetics, and favourable dye sorption in water and wastewater [25].

Zinc oxide (ZnO) has a wide range of uses as a low-toxicity material, including in the catalyst industry [26], gas sensors [27], solar cells [28], and medicine [29]. In addition, zinc oxide (ZnO) belongs to the class of metal oxides that are commercially very important due to their remarkable applications in various industrial fields, such as catalysis, solar cells, paints, UV light-emitting devices, electronic devices, biomedicine, and cosmetics. Likewise, ZnO NPs as semiconductors have gained attention for their wide range of applications, including optoelectronics, optics, electronics, and dye removal employing environmentally benign synthesis components, including fungi, bacteria, and marine macroalgae [30]. Additionally, ZnO has been discovered to be more effective, possibly beneficial, than other metals for the bio-synthesis of nanoparticles (NPs) for medical applications [31].

Zinc oxide nanoparticles (ZnO-NPs) are the most important of the metal oxide nanoparticles (MO-NPs) because of their unique chemical and physical features, which increase their applicability [32]. The elimination of several pollutants from the environment is a challenge, and adsorption methods are generally thought to be more facile and effective. Bearing a large theoretical specific surface area [33], the practical use of ZnO in water cleaning, including decontamination and reuse, has attracted excessive attention in recent years. As it contains additional functional groups, ZnO has shown higher adsorption performance. Therefore, ZnO might have more potential in adsorption technologies. In addition, ZnO was found to be more effective as an adsorbent than other absorbents, such as phosphate, iron oxide, and activated carbon, for the removal of sulphur compounds and H_2_S [34].

ZnO-NPs have recently been reported to efficiently absorb dyes from aqueous environments [35]. In addition, Ismate violet 2R was chosen as a model compound in the current study because of its broad application range, which includes colouring paper, dyeing cotton and wool, coating paper stock, and medicinal applications, as well as its potential harm.

In this work, a composite of zinc oxide nanoparticles was produced by chemical reduction using zinc nitrate as an adsorbent for the removal of Ismate violet 2R (IV2R) dye from aqueous solutions under various conditions by employing the batch adsorption technique. The initial dye concentration, pH, contact time, temperature, and adsorbent dosage were the main parameters assessed. Moreover, SEM, FTIR, UV, and BET methods were used to reveal the surface functionalisation, morphology, and pore size of the composite. In addition, the experimental equilibrium was applied using several different adsorption isotherm models to assess the adsorption mechanism, as well as thermodynamic and kinetic analyses. Furthermore, error functions were applied to reveal the most suitable model.

## 2. Materials and Methods

### 2.1. Materials

Polyvinyl pyrrolidone (PVP), H_2_O_2_, zinc nitrate, and sodium hydrosulphite were provided by M/s. Himedia Laboratories Pvt. Ltd., Mumbai, India. Table 1 shows the chemical structure of IV2R dye (molecular formula, molecular weight, and λ max). All of the used compounds were of analytical grade.

### 2.2. Synthesis of ZnO Nanoparticles

An improved approach was used to create ZnO-NPs, as described previously [26]. Briefly, 0.1 M Zn (NO_3_)_2_ was hydrolyzed with 250 mL of 0.2 M NaOH, and 1 % PVP was added and stirred continuously for 2 h using 100 mL of deionised water (Millipore, Milli-Q, Buenos Aires, Argentina). The pellet was produced by centrifuging the suspension at 3000 rpm for 5 min at 4 °C. At 75 °C, 1 M H_2_O_2_ was added and agitated for 1 h. The sample was also dried for 3 h at 65 °C in an oven before being maintained at 350 °C for 6 h.

### 2.3. Preparation of Dye Solution

The IV2R dye stock solution was made by weighing 1.00 g of powdered dye. The dye was transferred quantitatively into a 1 L measuring flask, which was then filled with distilled water to achieve a dye concentration of 1000 mg L^−1^ in the solution. The stock dye solutions (1000 mg L^−1^) were prepared separately and stored at 4 °C in distilled water. Dilution of the stock solutions with distilled water generated the working solutions.

### 2.4. ZnO-NP Characterisation

A scanning electron microscope was used to examine the morphologies of the adsorbents (JEOL JSM 6360 LA, Austin, TX, USA). Brunauer-Emmett-Teller (BET) desorption-adsorption studies were also performed using a sorptiometer (Quantachrome TouchWinTM v1.2, Downers Grove, IL, USA) and N_2_ adsorption isotherms. Using N_2_ desorption-adsorption measurements in a N_2_ solution with a saturated vapour pressure of 33.5 atm and an adsorption temperature of 77.3 K, the average particle radius, mean pore diameter, and pore volume of the ZnO-NPs were estimated. Furthermore, a Shimadzu FTIR-8400 S was employed to analyse the functional groups of the pre-prepared ZnO-NP materials using FTIR analysis spectrophotometry (Japan), and a UV-vis spectrophotometer was used to take spectrophotometric readings (UV 4000, MRI, Germany).

### 2.5. Adsorption Experiments

#### 2.5.1. Batch Adsorption Experiments

In a 100 mL airtight Erlenmeyer flask, batch studies for IV2R elimination were carried out with 50 mL of 10 mg L^−1^ dye solution. The temperature of the system was kept constant at 25 °C. During the experiment, a weighed amount of adsorbent was placed in the flask and mixed with various concentrations of IV2R at constant and moderate mixing rates. At predetermined contact times, samples were removed and filtered to separate the adsorbent from the dye solution. The concentration of the dye solution was assessed using spectrometric techniques, and all experiments were carried out in triplicate, with the mean result reported. Furthermore, the effects of contact time (15 to 180 min) and adsorbent dosage (0.005 to 0.08 g) were investigated at a constant initial dye concentration of 10 mg L^−1^. Experiments were conducted by altering the initial dye concentrations from 10 to 80 mg L^−1^. The temperature (25 to 55 °C) was evaluated at a constant dose of 0.02 g of ZnO-NP adsorbent to determine the effects of the initial dye concentration on dye uptake. Moreover, the effects of final pH on IV2R adsorption were investigated by altering the dye’s initial pH from 2 to 10. NaOH and HCl solutions were used to adjust the dye solutions’ initial pH. A UV-visible spectrophotometer was used to measure the quantity of IV2R dye in the clear supernatant at 550 nm at any time after shaking.

#### 2.5.2. Analytical Techniques

The dye concentrations were measured using a Spectronic Genesy 2PC UV-vis spectrophotometer at the wavelength of its maximum absorbance, λ max. Using the Beer-Lambert equation, the final dye concentration was measured spectrometrically to correspond to the dye’s maximum concentration:

Absorbance = ԐCSl(1)

where Ԑ is the molar absorptivity, CS is the sample concentration, and l is the thickness of the absorbing material (1 cm). A pH/ion meter (WTW Inolab pH/ION Level 2, Germany) was used to determine the pH of the dye solution. Equations (2) and (3) were used to calculate the adsorption capacity (q_e_) and dye elimination percentages [4]:

(2) $$q_{e}=\frac{\left(C_{i}-C_{e}\right)\times V}{W}$$

(3) $$\mathrm{Removal}\;\mathrm{Percentage}\;\left(\%\right)=(\frac{\left(C_{i}-C_{e}\right)}{C_{i}}\times 100)$$

where C_i_ and C_e_ (mg L^−1^) indicate the initial and final concentrations of IV2R at a given time, respectively, while V represents the volume of the dye mixture (L), and W represents the weight of the dry adsorbent (g).

### 2.6. Study of the Adsorption Isotherm

#### 2.6.1. Experiment with Isotherms

Experiments were carried out to develop adsorption isotherms for the ZnO-NP adsorbent by adjusting the adsorbent dose to 0.02 g at an initial dye concentration of 10 to 80 mg L^−1^ and an ambient pH of 2 for 3 h at 150 rpm, which was mixed with 50 mL of dye solution [36,37]. The following isotherm models were used to determine the most suitable one and calculate the data.

#### 2.6.2. Freundlich Model

The ability of the Freundlich model to fit the experimental data was used to calculate the slope of n and the intercept value of K_f_ by displaying a curve of log q_e_ with respect to log C_e_. By visualizing the Freundlich model in logarithmic form [38], it is simple to linearise it:

Log q_e_ = Log K_f_ + 1/n Log C_e_ (4)

The isotherm was used to determine the Freundlich constants K_f_ and n.

#### 2.6.3. The Langmuir Model

The Langmuir model is represented by the following mathematical formula [39]:

q_e_ = q_max_ bC_e_/(1 + bC_e_) (5)

where q_max_ (mg g^−1^) is the maximum sorption capacity corresponding to the saturation capacity, and b (L mg^−1^) is a coefficient relating to the affinity between ZnO-NP and IV2R dye ions. The linear relationship obtained by graphing the curve (1/q_e_) vs. (1/C_e_) is given in Equation (6).

1/q_e_ = 1/(bq_max_ C_e_) + 1/q_max_ (6)

The slope and intercept, respectively, are used to calculate b and q_max_.

#### 2.6.4. Henderson and Halsey Models

These models perform well with heteroporous substances and multilayer sorption. Using the equation below, the Halsey model [40] was calculated, as given in Equation (7).

(7) $$\mathrm{Ln}\;q_{e}=\frac{1}{n}\mathrm{Ln}\;K+\frac{1}{n}\;\mathrm{Ln}\;C_{e}$$

where n and K are Halsey constants. Meanwhile, the Henderson model was obtained from the following equation:

(8) $$\ln[-\ln(1-C_{e})]=\ln K+(\frac{1}{n})\ln q_{e}$$

where the Henderson constants are nh and Kh.

#### 2.6.5. The Harkins-Jura Model

This model describes multilayer adsorption, as well as the presence of heterogeneous pore scattering in an adsorbent [41]. This model was obtained from Equation (9).

(9) $$\frac{1}{q_{e}^{2}}=\left(\frac{B_{2}}{A}\right)-\left(\frac{1}{A}\right)\log C_{e}$$

where the isotherm constants are A and B.

### 2.7. Error Function Test

Various error functions were investigated in order to find the best and most appropriate model for investigating the equilibrium data. The error function tests were employed as follows:

#### 2.7.1. Hybrid Fractional Error (HYBRID)

Because it solves for low concentrations by balancing absolute deviation against fractional error and is more dependable than other error functions, the hybrid fractional error function was used. Equation (10) gives the hybrid error:

(10) $$HYBRID=\frac{100}{N-P}{\sum \left|\frac{q_{e,exp}-q_{e,calc}}{q_{e,exp}}\right|}_{i}$$

where *N* is the total number of data points, and *q_e,exp_* and *q_e,cal_* (mg g^−1^) are the respective experimental and calculated adsorption capacity values. In addition, *P* is the number of isotherm factors.

#### 2.7.2. Error Percentage Average (APE)

The APE model exhibits the suitability or trend between the predicted and observed values of the sorption capacity used to create model curves (APE) and can be designed using Equation (11).

(11) $$\mathrm{APE}(\%)=\frac{100}{N}{\sum_{i=1}^{N}\left|\frac{q_{e,isotherm}-q_{e,calc}}{q_{e,isotherm}}\right|}_{i}$$

where *N* is the number of data points under investigation.

#### 2.7.3. Nonlinear Chi-Square Test (Nonlinear Chi-Square Test)

The nonlinear chi-square test is a statistical method for determining which treatment system is most suitable. The following Equation (12) defines the approach to determining the chi-square error:

(12) $$X^{2}=\frac{{\left(q_{e,isotherm}-q_{e,calc}\right)}^{2}}{q_{e,isotherm}}$$

#### 2.7.4. Sum of Absolute Errors (EABS)

An increase in errors improves the fit, resulting in a bias toward high-concentration data. The following Equation (13) can be used to evaluate EABS tests:

(13) $$\mathrm{EABS}={\sum_{i=1}^{N}\left|q_{e,calc}-q_{e,isotherm}\right|}_{i}$$

### 2.8. Adsorption Kinetics

Adsorption kinetic experiments were carried out using 0.05 g of adsorbent mixed separately with 50 mL of IV2R solution containing 10 mg L^−1^ concentrations in 100 mL conical flasks at a solution pH of 2, and the mixture was agitated at room temperature for 15, 30, 60, 120, and 180 min. The clear solutions were examined for any remaining IV2R concentrations.

#### 2.8.1. Pseudo-First-Order Kinetics

The following equation gives the linear version of the generalised pseudo-first-order kinetics of dyes adsorbed at time t (mg g^−1^).

dq/dt = K_1_ (q_e_ − q_t_) (14)

The linear formula of the pseudo-first-order kinetics is expressed in Equation (15).

Log (q_e_ − q_t_) = log (q_e_) − k_1_t/2.303 (15)

where q_e_ is the amount of dye adsorbed at equilibrium (mg g^−1^), q_t_ denotes the amount of time t, and K_1_ denotes the pseudo-first-order rate constant (min^−1^).

#### 2.8.2. Pseudo-Second-Order Kinetics

The pseudo-second-order Equation (16) is as follows:

T/qt = 1/K_2_q_e_^2^ + t/q_e_ (16)

where K_2_ denotes the second-order rate constant (g mg^−1^ min). Plotting (t/q_t_) versus (t) yields a linear relationship, and the slope and intercept can be used to derive the q_e_ and K_2_ parameters, respectively.

#### 2.8.3. The Intraparticle Diffusion Model

The following is the intraparticle diffusion equation:

K_dif_ t^1/2^ + C = q_t_ (17)

where C is the intercept, and K_dif_ (mg g^−1^ min^0.5^) is the intraparticle diffusion rate constant, which is calculated from the regression line’s slope.

### 2.9. Thermodynamics of Adsorption

The value of Gibbs free energy change (ΔG°) is a fundamental principle of non-spontaneity, and a thermodynamic analysis is required to determine whether the nature of the sorption process is spontaneous or not. The following nonlinear forms (18)–(21) can be used to compute the Gibbs free energy change (ΔG° kJ mol^−1^), enthalpy change (ΔH° kJ mol^−1^), and entropy change (ΔS°, J mol^−1^ K^−1^) parameters for the sorption process at various temperatures (e.g., 25, 30, 45, and 55 °C):

K_d_ = q_e_/C_e_ (18)

ΔG° = −RT Ln K_d_ (19)

ΔG° = ΔH° − T ΔS° (20)

ΔG° = T (ΔS°) + ΔH°(21)

In addition to ΔG°, which can be obtained from Equation (20) or (21), the values of ΔH° and ΔS° were computed using the intercept and slope of the plotted curve of T vs. ΔG° from Equation (20) or (21).

## 3. Results and Discussion

### 3.1. ZnO-NP Characterisation

#### 3.1.1. Infrared Spectroscopy (FTIR)

**Figure 1:**
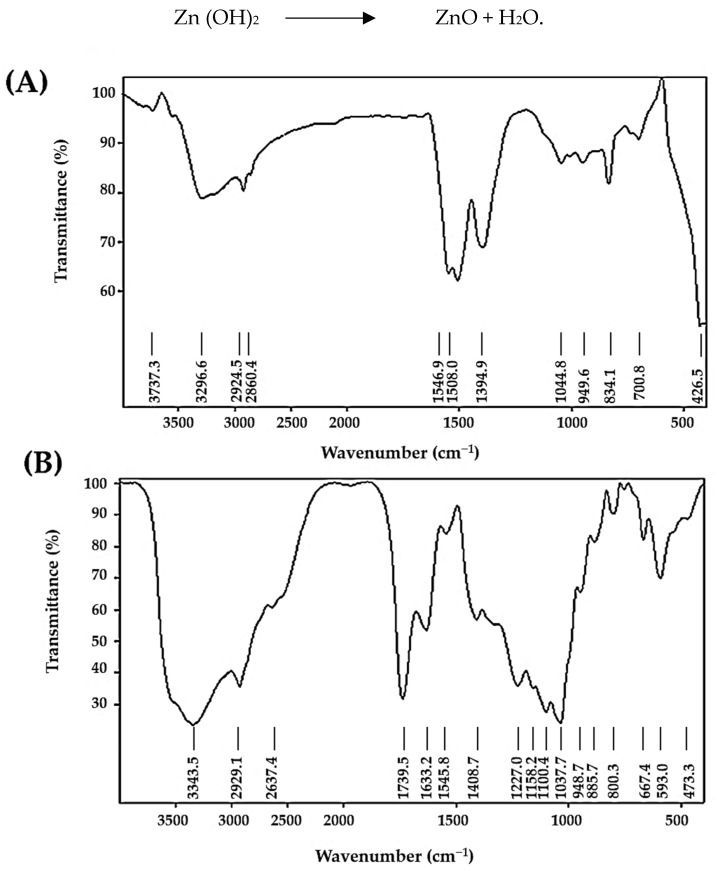
FTIR pattern of ZnO-NP before (**A**) and after adsorption of IV2R dye (**B**).

The produced ZnO-NPs were identified using FTIR measurements, as depicted in Figure 1. Figure 1A shows the existence of IR signals before adsorption in the range of 3600–3000 cm^−1^ in dye samples, which indicate the presence of –OH and –NH_2_ groups. Similarly, CH_2_ stretching vibration is indicated by the peak at 2935 cm^−1^. The stretching vibration of the C–H bond in –CH_2_ groups is attributed to the band at 2860 cm^−1^. According to the data, the peak at 1546 cm^−1^ is O–H bending vibration. Correspondingly, the peaks around 1394 and 1508 cm^−1^ indicate stretching of the C–H bond, which is attributed to CH_3_ vibration. Likewise, the absorption bands in the range of 1000–1300 cm^−1^ indicate the presence of the C–O group. In addition, the spectra of ZnO-NPs reveal a strong peak at 426 cm^−1^, which corresponds to the zinc-oxygen stretching mode [42]. The peaks at 949, 834, and 700 cm^−1^ generally correlate to C–H bending vibration.

On the other hand, Figure 1B shows that the bands at 3737, 3294, 2924, 2860, 1546, 1508, 1394, 1044, and 949 cm^−1^ were shifted to 3343, 2929, 2637, 1545, 1408, 1037, and 948 cm^−1^ after IV2R adsorption. According to this hypothesis, OH, C–H, C=C, and C=O groups may play a role in the adsorption of IV2R onto ZnO-NPs, while the C=O bond may be seen in the peaks between 1400 and 1500 cm^−1^ [43]. Additionally, the new peak at 1633 cm^−1^ is compatible with the C=O stretching of proteins [44]. C–H bending, C–O, or C–C stretching vibrations are represented by absorption bands that are located between 1100 and 1000 cm^−1^. The presence of an aromatic ring on the dye compound is indicated by the band seen at 885 cm^−1^, which is caused by aromatic C–H out-of-plane vibration [45]. Free O–H and N–H stretching vibrations are responsible for the robust and wide absorption band located at 3371 cm^−1^. While the observed band at 1408 cm^−h^ is related to the C–N stretching bond of amino acids, the band observed at a wavelength of 1037 cm^−h^ can be ascribed to C–O–C. Moreover, C–H bending vibration is responsible for the weak absorption band with a centre radius of 667 cm^−1^. Finally, the stretching vibrations of Zn-O are responsible for the peak range of 400–600 cm^−1^ [46].

It is well known that the production of ZnO particles from the hydrolysis of Zn^2+^ ions in aqueous medium is a complicated process. Many polyvalent cationic species can form when Zn^2+^ ions interact with OH ions, and their formation is significantly influenced by the pH of the solution. However, based on the pH, temperature, and synthesis processes, the precipitation of ZnO particles is usually defined by a development unit that can be either Zn (OH)_2_ or Zn(OH)^2^_4_ ions. The dissolution-reprecipitation mechanism has been proposed as a mechanism for the formation of ZnO from Zn(OH)_2_ [47]. ZnO will be produced by the chemical reaction [48]:

#### 3.1.2. BET Surface Analysis

**Figure 2:**
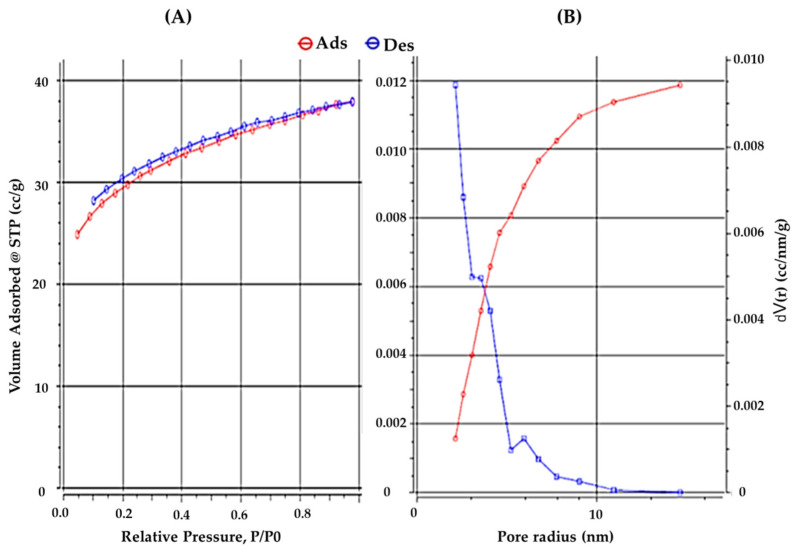
BET specific surface area (**A**) and adsorption-desorption isotherm examination (**B**) of ZnO-NPs.

The pore diameter, pore size distribution, and specific surface area of the ZnO-NPs were measured using BET and the nitrogen adsorption-desorption isotherm technique, as shown in Figure 2. It was revealed that an isotherm existed when the BET analysis was conducted at high P/P0 levels. Table 2 shows the BET results. The total pore volume and specific surface area of the ZnO-NPs were 95.83 m^2^ g^−1^ and 0.058 cm^3^ g^−1^, respectively. The specific surface area calculated using the Langmuir technique was 140.692 m^2^ g^−1^. This superior property could provide ZnO-NPs with a larger surface area, allowing more active sorption sites to occur. The micropore volume of ZnO-NPs was determined to be 0.015 cm^3^ g^−1^ using a cumulative BJH adsorption experiment. Furthermore, the average particle radius was 1.42 nm.

#### 3.1.3. SEM Examination

**Figure 3:**
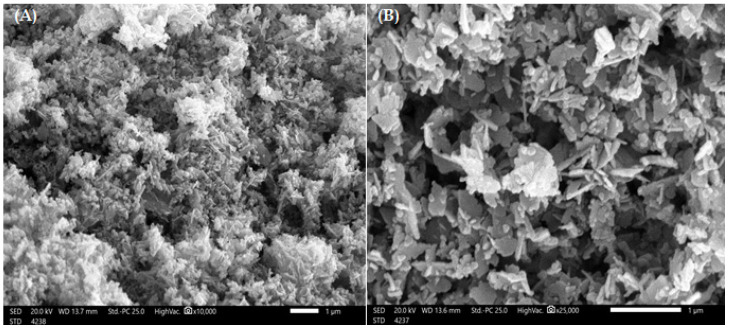
SEM examination at 1 µm for the prepared ZnO-NP at magnifications of 10,000× (**A**) and 25,000× (**B**).

SEM measurements are helpful in determining the surface morphology of the ZnO-NP structure. SEM pictures of ZnO-NP powders can be seen in Figure 3, which shows the particle morphologies. The nanostructure, which resembles nanoflowers and has agglomerated nanoparticles with an average pore size of approximately 1.22 nm, refers to ZnO-NP powders that result in formations that resemble flowers in uniformly sized nanoporous channels. The length and thickness of these formations are in nanometres. SEM micrographs of pure ZnO-NPs reveal that they have a porous character and a large surface area. These flowers and swollen structures are preferable for the absorption of dye contaminants.

#### 3.1.4. UV–Visible Spectra

The wavelength range of metal nanoparticles was determined by UV, and the results are shown in Table 3. The maximum absorbance is shown by the highest peak. The production of zinc oxide nanoparticles is confirmed by UV spectrometer absorption peaks in the 300–400 nm (390 nm) range. These peaks also indicate that the particles are nanosized and have a narrow particle size distribution. The absorption peak at 243 nm, which is associated with π→π * transitions in the sesquiterpene system, can hardly be seen in the UV spectrum of ZnO-NPs, which could be owing to a change in the sesquiterpene structure and the absence of these π→π * transitions [49].

### 3.2. Adsorption Experiments

#### 3.2.1. Influence of pH

**Figure 4:**
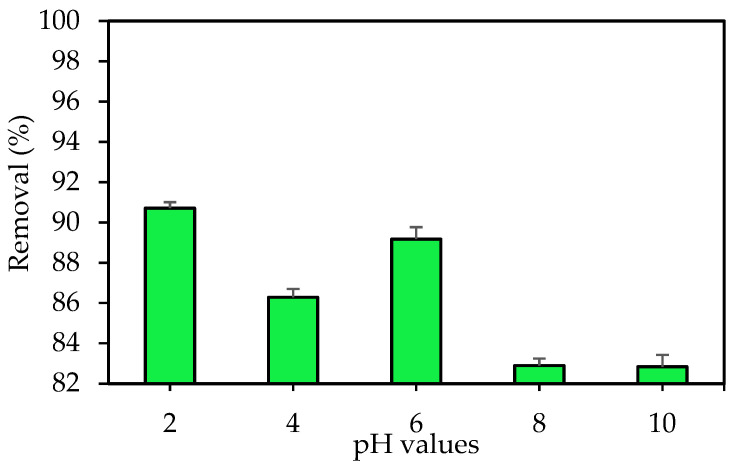
Influence of the pH value on the sorption of IV2R dye.

The shape and chemistry of the target dye ions, as well as the binding sites on the adsorbent, can be affected by the pH of a solution. In addition, the speciation of IV2R dye affects leads to an alteration in the reaction kinetics in addition to the equilibrium features of the sorption process [50]. Experiments were investigated by using different initial pH values of 2, 4, 6, 8, and 10 for the adsorbent to explore the effect of pH on the adsorption of IV2R. The percentage of adsorption tended to increase as the pH increased from 2 to 6. Furthermore, when the pH of the original dye solution was 2 and 6, the percentage of dye removal was at its maximum (90.7% and 89.17%, respectively) for the ZnO-NP adsorbent (Figure 4). The maximum adsorption capacity was observed at pH 2 and 6 with 4.31 and 4.24 mg g^−1^, respectively. These results indicate that solutions that are acidic in nature are efficient for the adsorption of IV2R dye when the adsorption is below pH 6. In addition, these results prove that IV2R removal was slightly decreased at pH 4 with a percentage removal of 86.28% and an adsorption capacity of 4.10 mg g^−1^. This result implies that there is a considerable electrostatic attraction between the adsorbent surface and the dye; this is likely owing to an increase in positively charged sites on the adsorbent surface when pH decreases, as reported by Netpradist et al. [51].

This study also revealed that anionic dye IV2R sorption is best in an acidic solution (positively charged adsorbent surface). Increased solution pH caused deprotonation and, as a result, a negatively charged surface of the adsorbent; consequently, this event may cause IV2R adsorption to decrease. Moreover, the nature of the surface charge of the adsorbent is affected by the pH of the solution in any adsorption system. In an acidic solution, the adsorbent’s oxide surface acquires a net positive charge. Therefore, anionic dyes have a stronger electrostatic attraction in acidic solutions than in basic media [52]. Other probable reasons for such observations include dye-adsorbent interactions owing to hydrogen bonding and hydrophobic-hydrophobic interaction mechanisms. Furthermore, the surface area and pore size, which remain unaffected by pH changes, play a significant role during the process [53,54].

Ghoneim et al. [55] found that at a higher pH, elimination is reduced in comparison to the maximum condition. This can be explained by the binding site’s ability to activate under normal conditions.

#### 3.2.2. Influence of ZnO-NP Dose

**Figure 5:**
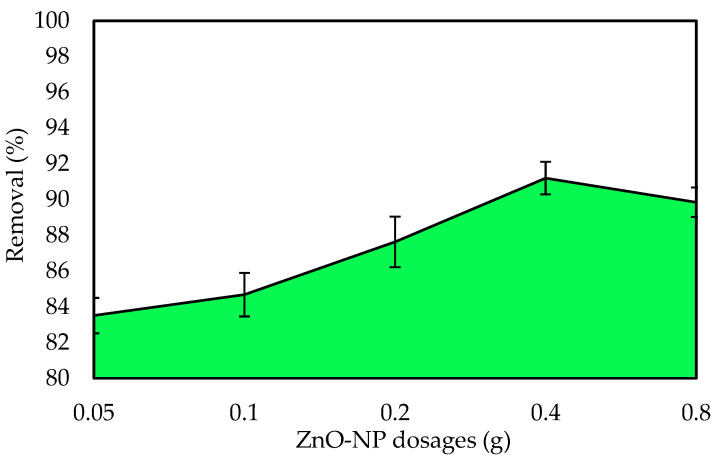
Influence of the ZnO-NP dose on the removal of IV2R.

The adsorbent dose is one of the most important factors for examining the impacts of the adsorption process to achieve the maximum adsorption capacity of the adsorbent by measuring the amount of ZnO-NP adsorbent. Experiments were performed with different adsorbent doses from 0.005 g to 0.08 g at a constant initial dye concentration of 10 mg L^−1^ to investigate the impact of the ZnO-NP dose on the adsorption system. At their respective equilibrium contact times, the percent adsorption increased from roughly 83.5 to 91.4 % as the adsorbent dose increased from 0.005 g to 0.04 g (Figure 5). It is self-evident that as the adsorbent dose increases, the dye uptake is enhanced. It is well established that as the adsorbent dosage increases, the % adsorption increases. The adsorbed amount per unit mass decreases. The amount adsorbed per unit mass was only 3.4 mg g^−1^ adsorbent when the dose was 0.08 g. The removal percentage was roughly 89%. The adsorption density decreases as the adsorbent dose increases due to unsaturated adsorption sites during the adsorption process [56]. Another cause could be intraparticle interactions, such as aggregation, as a result of a high adsorbent dose. The total surface area of the adsorbent is reduced because of this aggregation, while the diffusion path length rises [57].

#### 3.2.3. Influence of Contact Time

**Figure 6:**
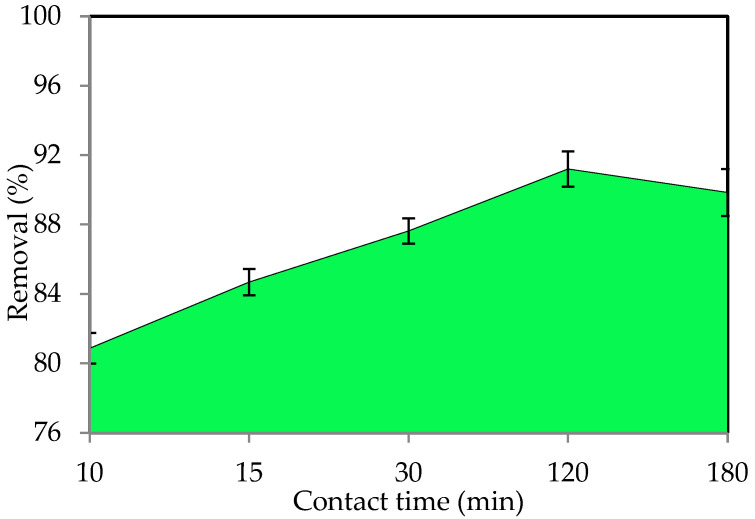
Effect of contact time on the removal of IV2R by ZnO-NPs.

The equilibrium time is essential when considering economical water and wastewater applications, and contact time is an important component in all transfer phenomena for the adsorption process. The adsorption process was studied to establish the best contact time between 15 and 180 min. Figure 6 shows the adsorption removal of IV2R dye by the ZnO-NP adsorbent. Equilibrium was reached, and the optimum contact time for IV2R dye was chosen to be 120 min for the sorbate-sorbent contact. The uptake of IV2R was observed to occur in two phases as a function of time. The first phase involved fast dye uptake during the first 10 min of sorbate-sorbent interactions, followed by a slow dye removal phase that lasted significantly longer (>120 min) until equilibrium was reached. The higher sorption value at the start of the process could be attributable to the abundance of active sites on the sorbent at this time. The sorption process becomes less efficient during the slower phase as these sites are gradually occupied [58]. The IV2R removal effectiveness by ZnO-NPs was found to be 88.9% (4.23 mg g^−1^) at 180 min. Two-stage sorption has been extensively documented in the literature, with the first being quick and quantitatively dominant and the second being slower and quantitatively insignificant [59].

Furthermore, Ananta et al. [60] reported that adsorption was rapid at the first contact time, which happens in the early stage of adsorption after a few minutes, since most of the binding sites are free and the adsorbent sites are empty, allowing the dye ions to bind quickly to the adsorbent. David and Joseph [61] mentioned that adsorption occurs quickly and is often controlled by diffusion from the majority of the solution on the surface. Moreover, Kumar and Gayathri [62] stated that adsorption increases as the contact time increases, which is possible due to a larger surface area of ZnO-NPs being available at the start and the exhaustion of the conversion of external adsorption sites, where the adsorbate (dye particles) is transported from the external to the internal sites of ZnO-NP adsorbent molecules.

#### 3.2.4. Influence of the Initial Dye Concentration

**Figure 7:**
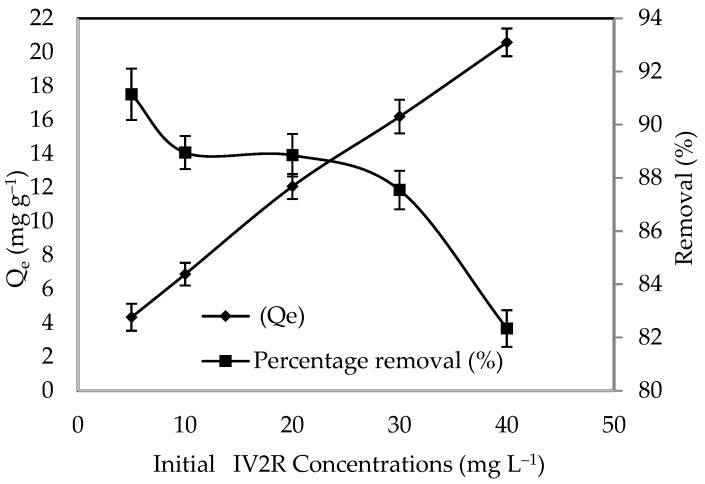
Influence of the initial IV2R dye concentration.

The amount of dye removed from an aqueous mixture is highly dependent on the dye concentration. At a constant temperature, the adsorption process for IV2R was examined at concentrations ranging from 10 to 80 mg L^−1^. The effect of IV2R dye concentrations on adsorption is seen in Figure 7. As can be seen, raising the initial dye concentration from 10 to 80 mg L^−1^ improved the ZnO-NP adsorption capacity from 4.33 to 20.58 mg g^−1^. This could be owing to the strong driving force that occurs when the initial concentration of the adsorbates is increased, overcoming the mass transfer resistance between the aqueous and solid phases [20]. Furthermore, the increased dye clearance at higher concentrations is most likely due to greater diffusion and decreased dye absorption resistance [63]. The availability of active sites on the adsorbent and the final occupancy of these sites are attributed to the rapid first stage of dye removal with this adsorbent; the sorption then becomes less efficient. In addition, the greatest uptake of IV2R dye was found at a concentration of 10 mg L^−1^ (91.14%). The equilibrium loading capacity and initial dye concentration have a strong linear relationship with correlation coefficients greater than 0.99 for the ZnO-NP adsorbent. A dye concentration of 10 mg L^−1^ was used for further experiments. In general, the decreasing percentage of dye removal with increasing dye concentration could be due to the increase in sorption sites on the adsorbent surface [64]. Furthermore, the high probability of dye ions colliding with the adsorbent surface and the high rate of dye ion diffusion onto the adsorbent surface could be linked to the large amount of dye adsorbed at a high dye concentration [65]. This finding could point to the potential of treating textile effluent with a higher dye concentration.

#### 3.2.5. Influence of Temperature

**Figure 8:**
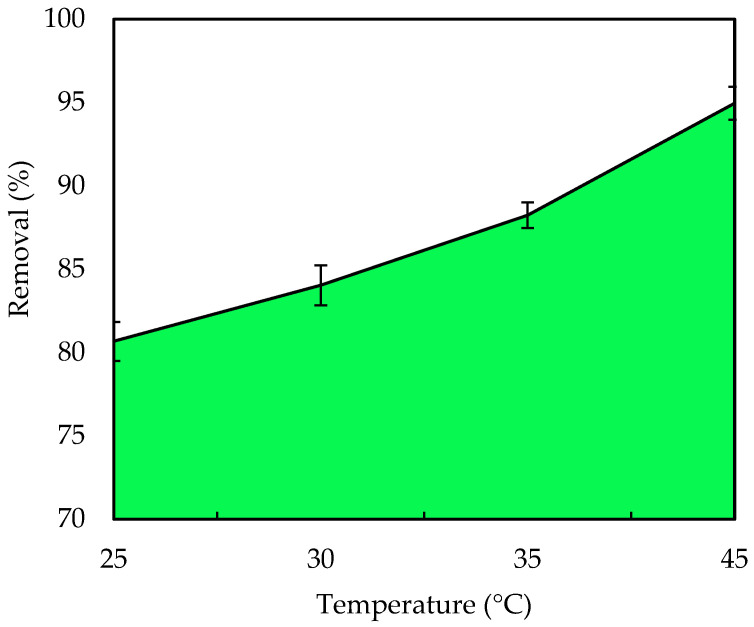
Effect of temperature on the removal of IV2R.

Figure 8 illustrates the percentage removal of IV2R dye ions by ZnO-NPs. The maximum percentage of dye removal was obtained at 45 °C with 94.9%, which rose when the temperature was increased from 25 to 45 °C for the IV2R dye on ZnO-NPs. Because of the decreased solution viscosity as the temperature rose from 25 to 45 °C, the diffusion rate of the adsorbate molecules within the pores changed, as did the equilibrium capacity of the ZnO-NPs for a specific adsorbate. Temperature increases (above 45 °C) resulted in a decrease in the percentage removed. This is attributed to a reduction in surface activity [66]. The decrease in adsorption efficiency can be attributed to a variety of factors, including deactivation of the adsorbent surface, a growing tendency for dyes to migrate from the solid to the bulk stage, and the destruction of specific active sites on the adsorbent surface due to bond ruptures. According to Sivaprakash et al. [67], this could be due to an increase in the mobility of the large dye ion as temperature rises. A growing number of molecules may be able to obtain enough energy to interact with active areas on the surface. Furthermore, rising temperatures may cause a swelling effect within the adsorbent’s internal structure, allowing large dyes to penetrate further. Additionally, there are two possible explanations for this outcome. At high temperatures, the pore diameters of adsorbent particles will expand [68]. Second, due to the breakage of some internal bonds, such as hydrogen bonds between the dye ion and the hydroxyl groups on the surface of the adsorbent’s active surface sites, the number of adsorption sites will increase [69,70].

### 3.3. Isothermal Analysis

The equilibrium isotherm is a plot of the quantity of sorbate extracted per unit sorbent (q_e_) as the sorbent’s solid phase concentration against the sorbate’s liquid phase concentration (C_e_). For the design and optimisation of an adsorption system for the removal of a dye from an aqueous solution, equilibrium isotherm data are necessary [71]. As a consequence, the most appropriate correlation for the equilibrium curve must be determined. To test the validity of the experimental data, a number of isotherm models were applied [22]. Therefore, in the present study, the most commonly used models, namely, the Langmuir, Harkins-Jura, Freundlich, Halsey, Henderson, and Tempkin isotherms, were used to describe the adsorption equilibrium.

#### 3.3.1. Freundlich Isotherm

**Figure 9:**
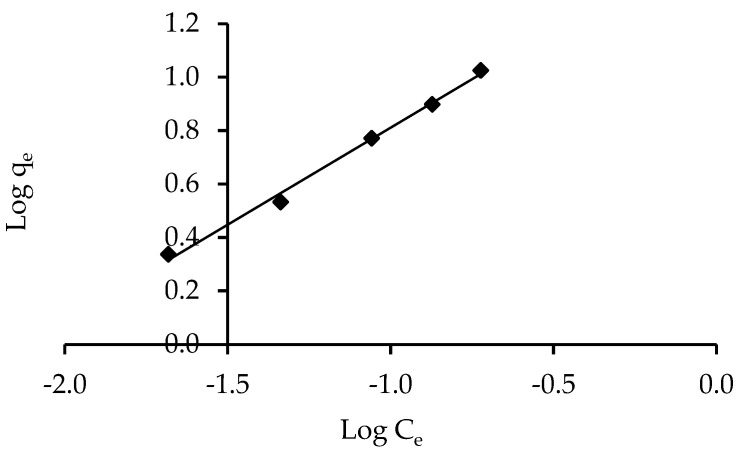
Freundlich isotherm of IV2R adsorption onto ZnO-NPs.

On the basis of the assumption of energy surface heterogeneity, the Freundlich isotherm model is the earliest known relationship describing non-ideal and reversible adsorption, which can be extended to multilayer adsorption. The obtained results were fit to the Freundlich isotherm model’s experimental data, which was supported by a strong correlation coefficient of R^2^ = 0.994 for the ZnO-NPs, showing that this model is beneficial for the adsorption process, as shown in Figure 9. The IV2R dye and the adsorbents formed a strong bond, as indicated by the value of 1/n, also known as the heterogeneity factor, which describes the divergence from sorption linearity as follows: When 1/n equals 1, the adsorption is linear, and the concentration of dye particles has no effect on the two stages. When 1/n is less than 1, chemical adsorption occurs; when 1/n is greater than 1, cooperative adsorption occurs, which is more physically advantageous and contains strong contacts among the adsorbate particles [72]. The value of the factor “1/n” was smaller than 1 in this study; the results indicate that a chemical sorption method on an external surface is preferable with this isotherm equation [22].

#### 3.3.2. Langmuir Isotherm

**Figure 10:**
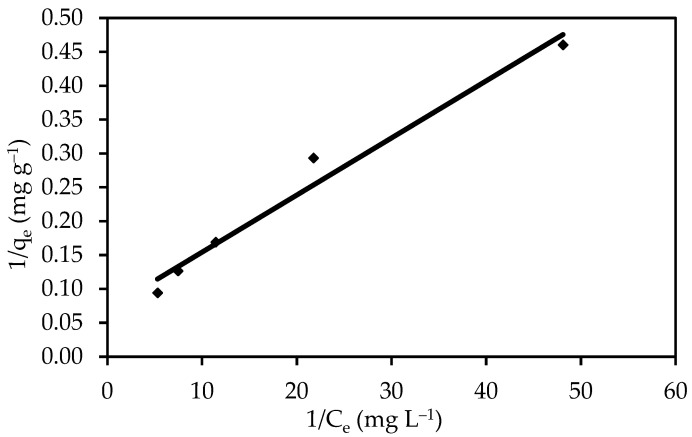
Langmuir isotherm for the sorption of IV2R.

The most popular model for quantifying the amount of adsorbate on an adsorbent as a function of partial pressure or concentration at a particular temperature is the Langmuir adsorption model. The Langmuir isotherm is based on the assumption of monolayer adsorption on a structurally homogeneous adsorbent, in which all adsorption sites are similar and energetically equivalent, adsorption occurs at specific homogeneous sites on the adsorbent, and once a dye molecule occupies a site, no further adsorption can occur. Table 4 shows the estimated parameters. The results obtained using the Langmuir isotherm coincided with data obtained throughout the experiment, with a strong correlation coefficient of R^2^ = 0.974 for the ZnO-NPs. In addition, the ZnO-NP dye has a high maximum absorption capacity (q_max_) of 119.05 mg g^−1^ (Figure 10). This is in line with the creation of a full monolayer on the adsorbent surface. The Langmuir constant (b), which is related to the heat of adsorption, was found to be 0.119. The dimensionless separation factor (R_L_), which is described below, can also be used to predict the affinity between the sorbate and the sorbent using Langmuir parameters (22):

(22) $$\mathrm{RL}=\frac{1}{1+\left(b*\mathrm{initial}\;\mathrm{concentration}\right)}$$

According to the parameters in Table 3, the value of R_L_ can be used to determine whether a sorption system is “favourable” or “unfavourable.” The sorption of IV2R onto ZnO-NPs has an R_L_ value of 0.597, which shows that adsorption of IV2R on ZnO-NPs was “favourable”.

#### 3.3.3. Harkins-Jura Isotherm

**Figure 11:**
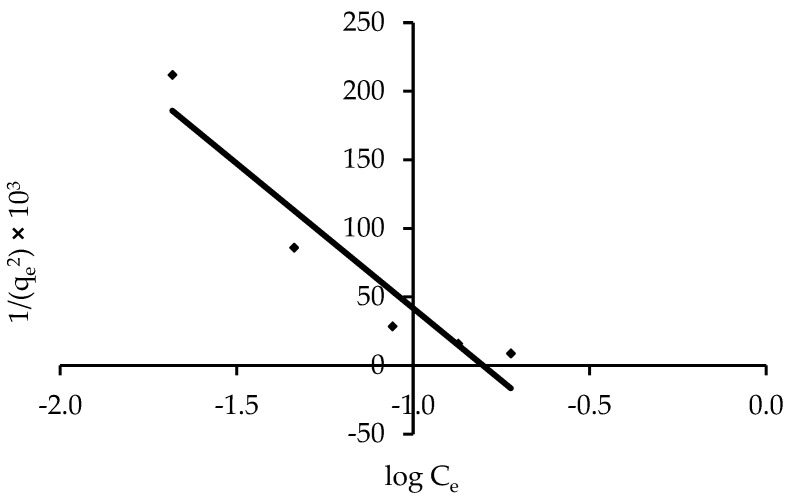
Harkins-Jura isotherm of the removal of IV2R on ZnO-NPs.

Multilayer adsorption is accounted for by the Harkins-Jura model, which can be explained by the existence of a heterogeneous pore distribution [41]. Equation (8) can be used to solve the Harkins-Jura adsorption isotherm, which can be seen in Figure 11 as a plot of 1/q_e_ vs. log C_e_. The presence of a heterogeneous pore distribution and multilayer adsorption can be explained by the Harkins-Jura model. The isotherm constants are presented in Table 4, and the correlation coefficient was found to be R^2^ = 0.905. This could mean that the Harkins-Jura model is useful for adsorption data.

#### 3.3.4. Isotherm Models of Halsey and Henderson

**Figure 12:**
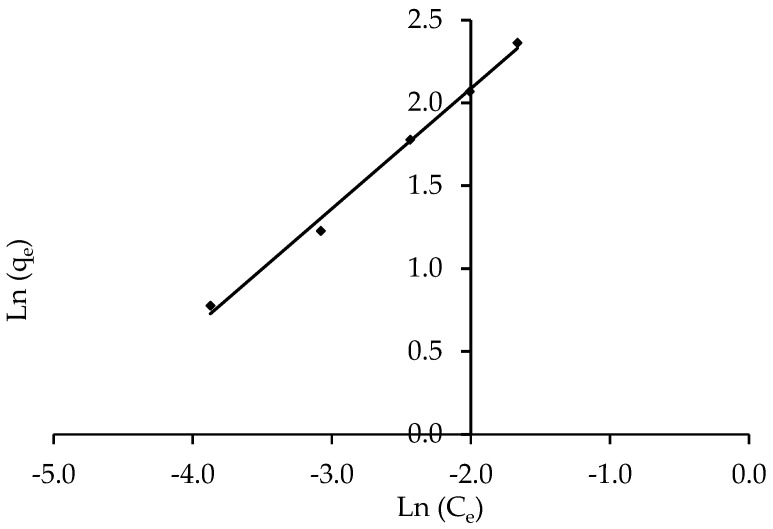
Halsey isotherm of the removal of IV2R by ZnO-NPs.

**Figure 13:**
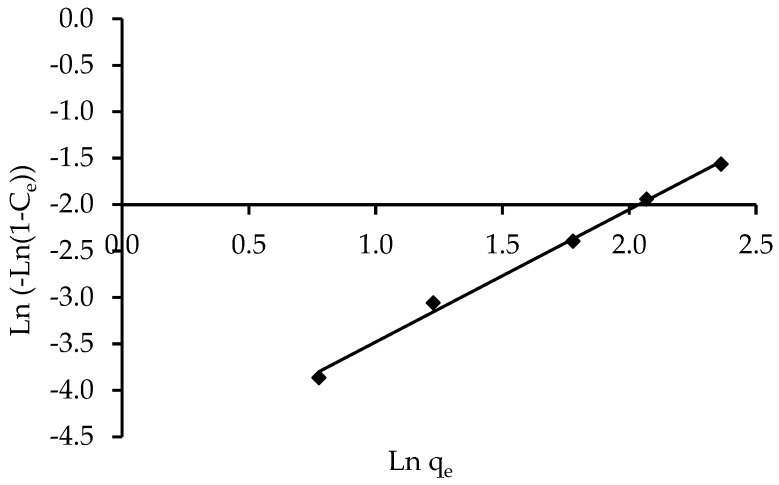
Linear Henderson isotherm of the removal of IV2R by ZnO-NPs.

The Halsey and Henderson isotherm models are suitable for multilayer adsorption, and the fitting of the Halsey equation can be applied to heteroporous solids [73]. Figure 12 and Figure 13 show plots of Ln q_e_ versus Ln C_e_ for Halsey and ln [ln(1C_e_)] versus Ln q_e_ for Henderson isotherms, respectively. Table 4 summarises the isotherm constants and correlation coefficients. The correlation coefficient for Halsey was R^2^ = 0.994, while Henderson’s was R^2^ = 0.995. The results of Halsey and Henderson suggest that both models can be used to predict IV2R adsorption on ZnO-NPs.

#### 3.3.5. Tempkin Isotherm

**Figure 14:**
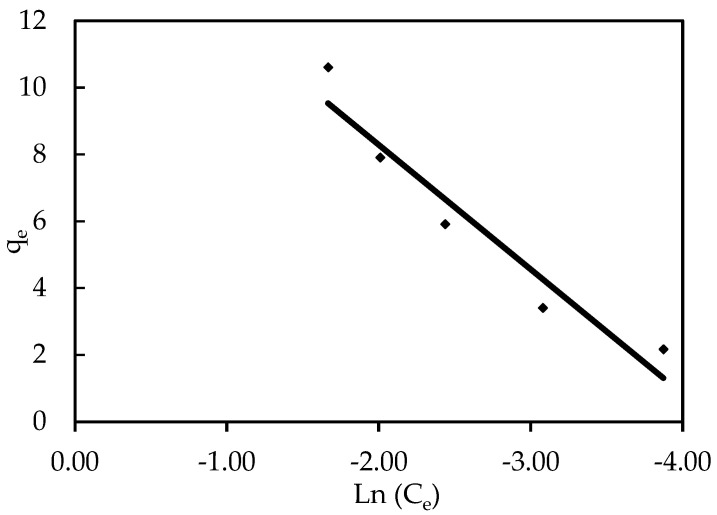
Tempkin isotherm of the removal of IV2R on ZnO-NP.

The Tempkin isotherm equation includes a component that describes the interactions between the adsorbing species and adsorbate [74]. It is assumed that due to adsorbate-adsorbate repulsions, the heat of adsorption of all molecules in the layer decreases linearly with coverage, and that adsorption is a uniform distribution of maximum binding energy [75]. The Tempkin adsorption isotherm model was used to evaluate the adsorption potentials of the ZnO-NPs for IV2R. The derivation of the Tempkin isotherm assumes that the fall in the heat of adsorption is linear rather than logarithmic, as implied in the Freundlich equation. The data indicate that the Tempkin isotherm model applies to the adsorption of IV2R dye onto ZnO-NPs, as shown by the high linear regression correlation coefficient (R^2^ = 0.928), as presented in Figure 14, which indicates that the Tempkin isotherm is appropriate for the equilibrium data attained for the adsorption of IV2R on ZnO-NPs.

### 3.4. Examining Error Functions to Find the Most Appropriate Isotherm Model

Several error functions, such as the hybrid fractional error, average percentage error (APE) equation, chi-square error (X^2^) equation, and the sum of absolute errors, were used to determine the best-fit model for the investigational data (EABS). Table 4 summarises the data gathered from the various error functions. For each isotherm model, the analysed error functions produced varying findings, and the comparison across isotherm models should focus on each error function independently [2]. Tempkin > Henderson > Freundlich > Langmuir > Halsey > Harkins-Jura are the most appropriate isotherm models based on the observed data. Nonetheless, the error function test offered variable data for all models, and the model evaluation focused on each error function individually.

### 3.5. Adsorption Kinetics

#### 3.5.1. Model of Pseudo-First-Order Kinetics

**Figure 15:**
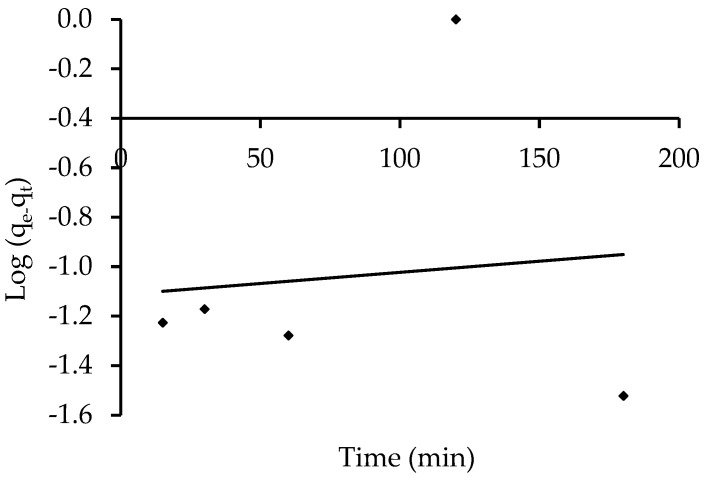
Adsorption kinetics of the pseudo-first-order kinetics of IV2R adsorption onto the ZnO-NPs.

The pseudo-first-order equation explains the adsorption rate based on the adsorption capacity. According to this model [76], the ratio of occupied to empty adsorption sites is proportional to the number of vacant sites. At a dye concentration of 10 mg L^−1^, the linear figure of log (q_e_–q_t_) versus t is shown in Figure 15. The intercept and the slope of the linear plots for the removal of the IV2R dye from ZnO-NPs were used to compute the q_e_ and K_1_ values. The values of K_1_, the experimental and calculated values of q_e_, and the correlation coefficients for the pseudo-first-order kinetic plots are provided in Table 5. The obtained experimental data do not agree with the theoretical values of q_e_. This implies that the kinetic data and the pseudo-first-order model do not match well. Moreover, because the correlation coefficients (R^2^) for the current experimental results for IV2R dye on ZnO-NPs were small, the pseudo-first-order equation was ruled out.

#### 3.5.2. Model of Pseudo-Second-Order Kinetics

**Figure 16:**
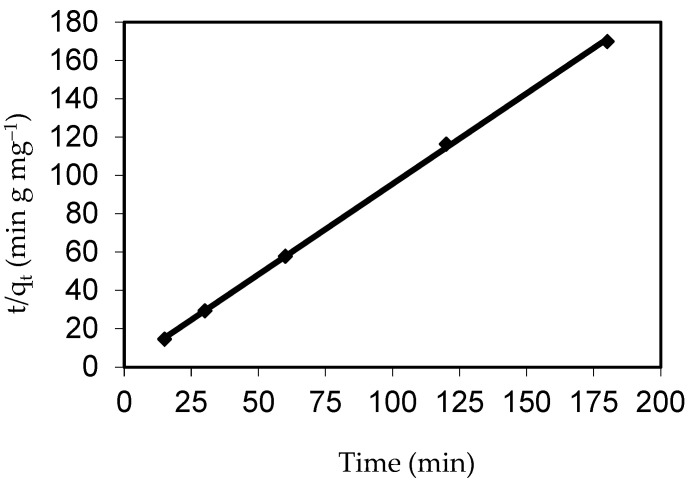
Adsorption kinetics of the pseudo-first-order kinetics of IV2R adsorption onto the ZnO-NPs.

The rate-limiting step is assumed to be due to chemical adsorption containing valence forces through the exchange and/or sharing of electrons between dye ions and the adsorbent in the pseudo-second-order equation (adsorbent) [76]. As shown in Figure 16 and Table 5, plotting (t/q_t_) against (t) should yield a linear correlation from which the data for parameters q_e_ and k_2_ may be determined from the slope and intercept, respectively. The second-order kinetic model’s correlation coefficient was more than 0.999, pointing to the fact that the pseudo-second-order kinetic model resulted in a good correlation for IV2R adsorption onto ZnO-NPs. Apart from that, it was clear that the k_2_ parameter value was higher than the corresponding k_1_ parameter value. This is because, according to David and Joseph [61], the adsorption rate is proportional to the square of the number of empty sites in the pseudo-second-order model.

#### 3.5.3. The Intraparticle Diffusion Equation

**Figure 17:**
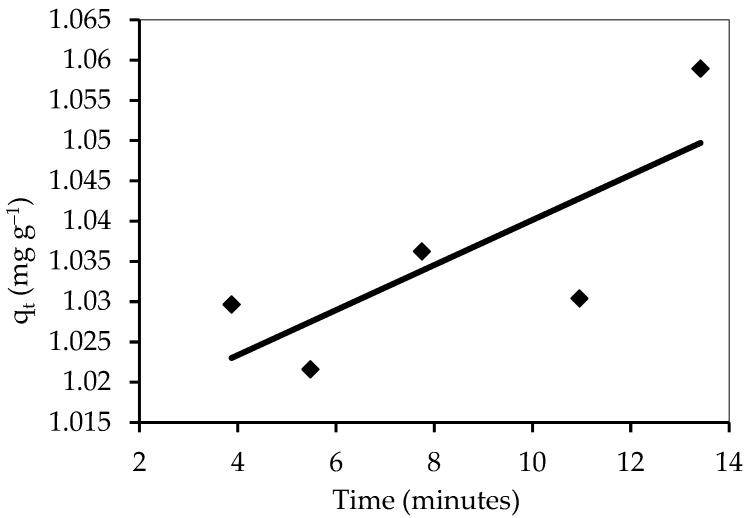
Adsorption kinetics of the intraparticle diffusion equation of IV2R adsorption onto ZnO-NPs.

The adsorption technique requires a number of steps, including the transport of solute particles from the aqueous part to the exterior of the solid molecules, followed by the diffusion of solute molecules into the cavities’ interior decoration, which is likely to be a slow process and a rate-determining step [77]. The figures of q_t_ inverse t^0.5^ may indicate a multilinear correlation, indicating that the adsorption process occurs in two or more stages (Figure 17 and Table 5). The slope directly estimates the rate constant K_dif_, and the intercept is C, as shown in Table 4. Because the barrier to exterior mass transfer increases as the intercept increases, the value of the C factor provides information about the thickness of the border layer.

### 3.6. Adsorption Thermodynamics

Table 6 shows the thermodynamic parameters of IV2R adsorption onto ZnO-NPs. The large negative value of ∆G^o^ demonstrates that dye sorption was spontaneous and feasible. Nonetheless, the free energy values in Table 6 increase with increasing temperature, demonstrating that the adsorption method is endothermic [78,79] and suggesting that the adsorption method’s spontaneity decreases at lower temperatures. For physisorption, the range of ∆G^o^ values is between −20 and 0 kJ mol^−1^, whereas, for chemisorption, the range is between −80 and −400 kJ mol^−1^ [3]. The activation energy measurements in this work indicate that IV2R sorption onto ZnO-NPs occurs via a physisorption process.

### 3.7. Application to Real-Life Wastewater

To test the validity of using ZnO-NPs as adsorbents, real wastewater was mixed with simulated dye samples to see if the adsorbent could remove IV2R under optimum conditions. The results revealed that changing the kind of water had a significant impact on dye removal, with deionised water having the least impact on the adsorption process, with 91.51% of dye removed at an acidic pH after 180 min. On the other hand, real wastewater contains very high quantities of interfering ions from a variety of contaminants, which had a major impact on the IV2R dye removal effectiveness, with 62.6% of dye removed after 180 min. Regardless, the results show that ZnO-NP adsorbents may be employed successfully to remove IV2R dye from aqueous mixes and wastewater at a reasonable cost.

### 3.8. Comparative Studies of ZnO-NP Sorption Capacity

To demonstrate the efficacy of ZnO-NPs, the results of this experiment were compared to those of other studies on the adsorption capabilities of various dyes. This adsorption capacity of ZnO-NPs for IV2R was also found to be significantly higher in comparison with some other recently reported adsorbents, as reported in Table 7.

## 4. Conclusions

SEM, FTIR, UV, and BET surface analyses were used to investigate ZnO-NPs. The presence of functional groups such as N–H, O–H, CH_2_, C–O, and Zn–O stretching was revealed by FTIR, which increased their ion-exchange capabilities for the selective adsorption of oppositely charged molecules. The total pore volume and specific surface area of the ZnO-NPs were 95.83 m^2^ g^−1^ and 0.058 cm^3^ g^−1^, respectively. The specific surface area calculated using the Langmuir technique was 140.692 m^2^ g^−1^. Likewise, ZnO-NPs are agglomerated nanoparticles with a size of 1.22 nm, similar to nanoflowers. The optimal operational parameters were found to be 10 mg L^−1^ IV2R with 0.04 g of ZnO-NPs and 60 min contact time at pH 2 and 45 °C. Moreover, the Halsey, Freundlich, Langmuir, Harkins-Jura, Henderson, and Tempkin models, on the other hand, were used to investigate the equilibrium isotherm adsorption results. The Langmuir model produced a greater adsorption capacity (q_max_) of 119.05 mg g^−1^. Different error function models were used to find the best-fitting isotherm model in this study. In addition, the intraparticle diffusion, pseudo-first order, and pseudo-second-order models were used in a kinetic investigation. The pseudo-second-order model effectively represented experimental data pertaining to the system under study with R^2^ = 0.999. Furthermore, the percentage of dye removal by ZnO-NPs from real wastewater was 65 %. Additionally, the thermodynamic parameters (∆G°, ∆H°, and ∆S°) of the sorption processes were estimated. IV2R dye adsorption was endothermic and spontaneous, and the adsorption reaction was a physisorption reaction. This technique takes advantage of the ability to remove a large quantity of dye in a short amount of time with a small amount of adsorbent.

## Figures and Tables

**Table 1:** The chemical and physical properties of the ISMATE 2R dye [2].

Features	Data
Dye name	ISMATE violet 2R
Mol. wt.	700
Molecular formula	C22H14N4O11S3CuCl
Wavelength (λ max)	550 nm
Molecular structure	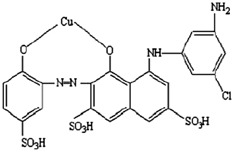

**Table 2:** Physicochemical properties of ZnO-NPs.

Characteristics	Value/Unit
Density	2.2 (g cm^−^^3^)
Langmuir method	140.692 m² g^−1^
BJH adsorption	10.682 m² g^−1^
BJH desorption	8.847 m² g^−1^
BET surface area	95.838 m² g^−1^
Average pore size	1.228 nm
Total pore volume	0.058 C2 g^−1^
BJH adsorption cumulative micropore volume	0.014 C2 g^−1^
Average particle radius	1.422 nm

**Table 3:** UV-vis spectra of chemical ZnO-NPs.

Wavelength (nm)	Abs.
390.50	0.100
243.000	0.528
380.51	0.350
500.86	0.537

**Table 4:** Parameters and error function of the isotherm models for IV2R removal by ZnO-NPs.

Isotherm Model	Isotherm Parameters	Value	EABS	X^2^	APE (%)	Hybrid
Freundlich	*1/n*	0.725	1.87	0.116	0.1173	0.343
	*n*	1.37				
	*K_F_* (mg^1−1/n^ L^1/n^ g^−1^)	34.30				
	*R* ^2^	0.994				
Langmuir	*Q_max_* (mg g^−1^)	119.05	36.21	71.84	7.934	312.359
	*b*	0.119				
	*R_L_*	0.597				
	*R* ^2^	0.974				
Harkins-Jura	*A_HJ_*	0.0047	762.50	754.6	3.95	3281.2
	*B_HJ_*	0.80				
	*R* ^2^	0.905				
Halsey	*n*	1.379	740.50	711.75	3.845	3094.5
	*K_H_*	131				
	*R* ^2^	0.994				
Henderson	*1/n_h_*	1.426	0.178	0.000	0.024	0.005
	*K_h_*	0.007				
	*R* ^2^	0.995				
Tempkin	*A_T_*	68.03	0.104	0.000	0.014	0.002
	*B_T_*	3.72				
	*b_T_*	255.5				
	*R* ^2^	0.928				

**Table 5:** Parameters of the different adsorption kinetic models.

Kinetic Models	Parameters	Value
First-order	q_e_ (calc.) (mg g^−1^)	12.94
	k_1_ × 10^3^ (min^−1^)	2.07
	R^2^	0.011
Second-order	q_e_ (calc.) (mg g^−1^)	1.06
	k_2_ × 10^3^ (mg g^−1^ min^−1^)	818.54
	R^2^	0.999
Intraparticle diffusion	K_dif_ (mg g^−1^ min^−0.5^)	0.0028
	C cal (mg g^−1^)	1.01
	R^2^	0.596

**Table 6:** Thermodynamic factors of the sorption of IV2R onto ZnO-NPs.

Temperature (°C)	∆G° (kJ mol^−1^)	∆H° (kJ mol^−1^)	ΔS° (kJ mol^−1^)
25	−12.98629244	47.92	−0.202
30	−12.8458117		
35	−12.81290783		
45	−20.94418521		
55	−16.5176115		

**Table 7:** Summary of the elimination of several dyes from aquatic mixtures by various ZnO-NPs.

ZnO-NP.	Dye Adsorbed	q_e_ (mg g^−1^)	Ref.
ZnO-NPs-AC	Acid yellow 119	116.29	[80]
AC-ZnO	Acid orange 7	32.13	[81]
AC-ZnO	Methylene blue (MB)	32.22	[81]
ZnO-NR-AC	Bromophenol red	200	[82]
ZnO-NP-AC	Malachite green	322.58	[83]
ZnO	Malachite green	310.50	[84]
ZnO-NRs-AC		113.64	[85]
Chloroacetic Acid-Modified Ferula	Basic dye	354.89	[86]
ZnO-NP	IV2R	119.05	Present study

## Data Availability

The data are available from the corresponding author upon reasonable request.
